# *“We do not know how to screen and provide treatment*”: a qualitative study of barriers and enablers of implementing perinatal depression health services in Ethiopia

**DOI:** 10.1186/s13033-021-00466-y

**Published:** 2021-05-05

**Authors:** Abel Fekadu Dadi, Emma R. Miller, Telake Azale, Lillian Mwanri

**Affiliations:** 1 College of Medicine and Public Health, Flinders University, Health Sciences Building, Sturt Road, Bedford Park, Adelaide, SA 5042 Australia; 2 Department of Epidemiology and Biostatistics, Institute of Public Health, College of Medicine and Health Sciences, University of Gondar, Gondar, Ethiopia; 3 Department of Health Education and Behavioural Sciences, Institute of Public Health, College of Medicine and Health Sciences, University of Gondar, Gondar, Ethiopia

**Keywords:** Perinatal depression, Health system, Ethiopia

## Abstract

**Background:**

Qualitative studies evaluating maternal mental health services are lacking in Ethiopia, and the available evidence targets severe mental illnesses in the general population. We conducted a qualitative study to explore barriers to, enablers of, or opportunities for perinatal depression health services implementations in Ethiopia.

**Methods:**

We conducted a total of 13 face to face interviews with mental and maternal health service administrators from different levels of the Ethiopian healthcare system. We interviewed in Amharic (a local language), transcribed and translated into English, and imported into NVivo. We analysed the translated interviews inductively using thematic framework analysis.

**Results:**

The study identified: (i) health administrators’ low literacy about perinatal depression as individual level barriers; (ii) community low awareness, health-seeking behaviours and cultural norms about perinatal depression as socio-cultural level barriers; (iii) lack of government capacity, readiness, and priority of screening and managing perinatal depression as organisational level barriers; and (iv) lack of mental health policy, strategies, and healthcare systems as structural level barriers of perinatal mental health implementation in Ethiopia. The introduction of the new Mental Health Gap Action Programme (mhGap), health professionals’ commitment, and simplicity of screening programs were identified enablers of, or opportunities for, perinatal mental health service implementation.

**Conclusions:**

This qualitative inquiry identified important barriers and potential opportunities that could be used to address perinatal depression in Ethiopia. Building the capacity of policy makers and planners, strengthening the mental healthcare system and governance should be a priority issue for an effective integration of maternal mental health care with the routine maternal health services in Ethiopia.

## Background

Depression is one of the most common complications of the perinatal period, both in high [1] and low-income countries [2]. In Ethiopia, the prevalence of antenatal depression ranges from 11 to 31% [3], while the prevalence of postnatal depression ranges from 12 to 33% [4, 5]. Untreated depression during pregnancy has been reported to increase the risk of obstetric complications [6], poor foetal growth [7, 8], and adverse birth outcomes [1, 2]. Similarly, untreated postnatal depression is reported to increase the risk of adverse infant health outcomes [9, 10] and their future educational achievement [11]. The negative impacts of perinatal depression on family and social disruption have been reported in different studies [4, 12, 13].

There have been efforts to mitigate the substantial burden of perinatal depression in developed countries, but much remains to be done in low-and middle-income countries [14–18]. In terms of access, mental health services in Ethiopia are numerically limited, geographically inaccessible, and hospital-based [19]. The treatment gap for postnatal depression is near to 95% [20], and, while unknown at this stage, it is expected to be similar for antenatal depression. Barriers to poor treatment are multifactorial and could be associated with personal behaviour [21, 22], the severity of the disorder [20], social norms [23], and the lack of effective mental health care systems [24]. There is a global initiative to reach women with mental disorders by integrating mental and maternal health services [25, 26]. However, situational analysis in five low- and middle-income countries showed a limited capacity of the health systems regarding feasible detection and treatment strategies [27].

In order to provide the right maternal mental health care where and when it is needed, it is critical to integrate maternal mental health services into the existing health system [28]. Appropriate service delivery, human resources for health, information, health technologies, budget, and governance structure are fundamental to building an effective health system [29]. Two programs have been running in Ethiopia to scale up maternal mental health services: (i) A Program for Improving Mental Health care (PRIME), which aims to generate evidence on the implementation and scale-up of mental health service integration [30]; and (ii) The Emerging mental health system in low-and middle-income countries (Emerald), which aims to improve mental health outcomes by generating capacity and evidence [31]. The Emerald program focuses on identifying health system barriers and developing solutions to improve the effective delivery of mental health services. Although there have been some quantitative studies focusing on severe mental illnesses in the general population in Ethiopia [19, 32, 33], there is a lack of qualitative studies that have investigated maternal mental health system and identified barriers to effective care of antenatal depression. In-depth information provided by qualitative approaches would help to identify gaps in policy, program, and health system that need to be addressed for successful integration of mental health services into routine maternal health services. This qualitative study explored health administrators’ and community perspectives of the health system’s response, barriers and enablers for effective perinatal depression care service delivery in Ethiopia.

## Methodology

### Theoretical framework

We used Smith’s et al. [34] Multilevel Conceptual Framework for barriers hindering maternal mental health services to aid the analysis, synthesis and summarizing of the study findings. The model posits that mental health service delivery is affected at individual, organizational, sociocultural, and structural level. Individual level barriers include factors such as knowledge, attitude, and behaviour of the community, health professionals, and health administrators working at different levels. The sociocultural related barriers include language, cultural values of the community, and women’s perceptions of perinatal depression. The organizational-level barriers include the capacity and readiness of the health facilities or organizations to provide maternal mental health services. This might have elements such as lack of resources (trained workforce, money), time (patient load), space (lack of adequate offices), lack of clarity in role and responsibilities, lack of working manuals, screening tools, treatment guidelines, and protocols. Finally, structural level barriers include lack of policy, program, and strategies, low attention and initiation by the government, and lack of transparent system and structure.

### Study setting and recruitment of study population

Ethiopia, a country located in the Eastern part (horn) of Africa, is administratively subdivided into nine regional states and two central cities [35]. The Ethiopian Federal Ministry of Health (FMOH) is mandated to formulate national policies, plans and programs for the health sector. Under FMOH, all regions have health bureaus, zonal health offices, hospitals, and health centres. Health structures under FMOH are centrally controlled, and all plans and programs cascade to the lower level of the health system. In the Ethiopian health care system, mental health service delivery involves the two wings of the healthcare system structure, health administrative wing that includes health offices from the federal to the district level health offices and health service delivery facilities such as hospitals and health centres. In health service delivery facilities, perinatal women can directly visit the psychiatry clinics for mental health services. Alternatively, they can visit the maternal and child health (MCH) clinics for routine follow up where they might be screened for mental health problems. Similarly, in health administrative wing, the mental health care team and MCH care team are involved in perinatal mental health service delivery. Health administrators from different levels of the healthcare system were interviewed to explore the barriers to and enablers of perinatal depression health services in Ethiopia.

We purposely sampled health administrators from different levels of the Ethiopian healthcare system focusing in Amhara Regional State [36, 37], to explore their perspectives and gain an in-depth understanding of perinatal depression care service delivery. The aim was to recruit study participants who had been closely involved in the healthcare system and can provide better opportunity to potentially explore the barriers to and enablers of perinatal depression health services. We interviewed a total of 13 participants who were leaders at different levels in mental health or maternal health services. The number of participants interviewed was determined by information saturation [36, 38]. As the health system policy, programs and guidelines are controlled centrally, all regions and health administrators working at different level would have the same level of experience in implementing perinatal depression health services as they are all governed by a common set of guidelines. So, it was practical and justifiable to reach information saturation with this small study participants.

### Recruitment of participants

**Fig. 1:**
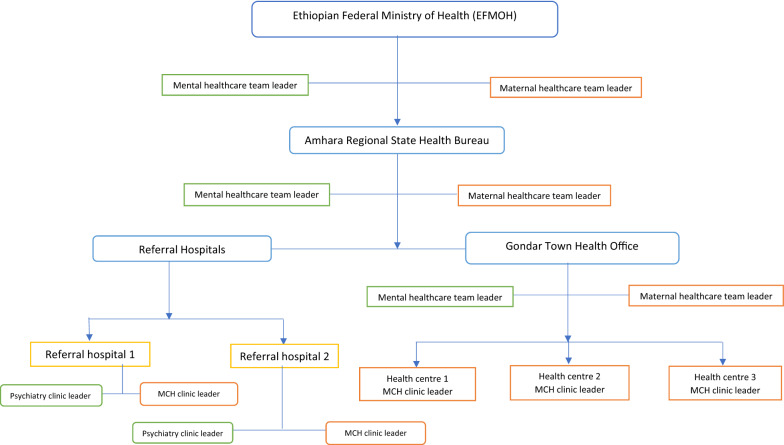
Study participants’ recruitment procedure for the qualitative study, 2017–2020, Gondar town, Ethiopia

Figure 1 (in supplementary information) shows the recruitment strategy we used at each level of the healthcare system. In the Ethiopian healthcare system, in health facilities, a pregnant woman can directly visit the psychiatry clinic for mental health services or maternal health clinics for perinatal services. As such, women with depression symptoms might present at both clinics. Therefore, health administrators working in the two clinics (maternal and psychiatry) from two referral hospitals were involved. Administratively, the maternal health care team and the mental health care team could be involved in perinatal mental health service delivery. Therefore, health administrators working as mental health and maternal healthcare team leaders at different levels of the Ethiopian healthcare system were involved. The remaining three participants were health professionals leading maternal and child health teams in three health centres.

### Data collection

We used a pre-tested semi-structured interview guide prepared in English and translated to Amharic (local language of the data collection area) to collect the data. The interview guide was developed based on literature related to the main research question. The interview guide developed in the way that it captures health service administrators’ knowledge, attitude, experience, and health system readiness to handle perinatal depression health services. In addition, we used probes, cues and prompts that directed the interviewees to focus on areas relevant to the research topic to enable collection of relevant and in-depth data [39]. The principal investigator (AFD) interviewed each participant face-to-face in their private offices (which were quiet, secure and comfortable) to maintain the quality of the recordings and facilitate open discussion [40–42]. The interviews were audio-recorded and took 15–35 min, with a summary of important points made at the end to make sure that the responses were correct and fully documented.

### Ethical consideration

The research was approved both by the Social and Behavioural Research Ethics Committee of the Flinders University, Australia (reference number 7959, 2018) and the Institutional Review Board of the University of Gondar (reference number O/V/P/RCS/05/1601, 2018). Study participants were informed about the purpose, objectives, and their right to decline participation or withdraw from participation at any time. Informed consent was obtained from each participant. Privacy and confidentiality were maintained throughout the study.

### Data analysis

We transcribed and translated the audio-recorded interviews verbatim, imported the data into NVivo software [43] and applied a thematic framework analysis [44]. We used a semi-structured interview guide to collect the data and a thematic framework analysis is found to be the best fit for analysis. The analysis involved the following steps: data familiarisation, coding, identification of a thematic framework, indexing, charting and data interpretation. Data familiarization occurred through the processes of transcribing and repeated listening to audio tapes, which helped to systematically identify initial ideas for coding [40]. After coding the first two transcripts, a thematic framework was developed by the research team for indexing the remaining transcripts. Indexing in NVivo is accomplished by adding concepts and ideas from the transcripts to the related codes and categorise created in the identified thematic framework. We used both inductive and deductive approaches to account for categories that were known a priori and those that originated from the data [45]. A framework matrix that clearly outlined the themes, sub-themes, and their description with their respective files and references was developed. Finally, the emerged themes, sub-themes with their clear descriptors were organized, presented and interpreted for meaning. Each theme and sub-themes with their central meaning were described and presented supported by quotes obtained from the study participants [45–48].

## Results

### Characteristics of the study participants

Socio-demographic characteristics of the participants are described in Table 1. A total of 13 participants were involved in this study, nine of whom were male (69%). The median age of participants was 34 years (range 26–55 years) and the median work experience was 11 years (range 4–35 years). Participants’ professional backgrounds were varied, with midwifery [6] and public health [4] being the two largest groups.

Three main themes and 6 sub-themes were identified that best explained our research questions. These are described in Table 2.

**Table 1:** Characteristics of the interview participants involved in a qualitative study, Gondar Town, Ethiopia (N = 13)

Participant characteristics	Participants(N = 13)
Median age (range)	34 (26, 55)
Sex	
Male	9
Female	4
Median work experience in the health system (range)	11 (4, 35)
Profession	
Midwifery	6
Psychiatrists	2
Psychologist	1
Master of public health	4
Place of work	
Health offices	6
Hospitals	4
Health centres	3

**Table 2:** Summary of barriers to and enablers of perinatal depression health services implementations in Ethiopia, 2018 (N = 13)

Themes	Sub-themes
Understanding about perinatal depression	1. Conceptualising perinatal depression 2. Management of and interventions for perinatal depression 3. Consequences of untreated perinatal depression 4. Perceptions on community awareness about perinatal depression
The health care system	1. Perinatal mental health policy and health care system 2. Government capacity, readiness, and prioritisation of perinatal depression
Enablers and opportunities for perinatal depression screening services	

### Understanding about perinatal depression

Seven key sub-themes emerged from the first theme, explaining the roles that health administrators and community mental health awareness and cultural issues played as barriers to implementing perinatal depression health services in Ethiopia.

### Conceptualising perinatal depression

Some administrators related the concept of perinatal depression to the WHO definition, which proposes health as a state of complete physical, mental and social well-being and not merely the absence of disease or infirmity [49]. One health administrator interviewed said:

A mother is said to be healthy as defined by the WHO: if she can use her mind properly, if she can resist for any source of stressors, if she is fruitful in any work or activity, and if she can manage or administer her family properly. In line to the WHO definition, health is not merely the absence of disease but should also include mental wellbeing. (Male, aged 32 years)

Other participants used a range of indicators to define maternal mental health: (i) adhere to perinatal follow-up and usual activities; (ii) overcome challenges (e.g. adjust or withstand life stressors or events related to parenthood); (iii) be confident about herself and her pregnancy (e.g. she has positive thoughts, she is happy and feels healthy during pregnancy or after birth); (iv) have good social and personal interactions.

So, if the mother is mentally healthy, she should be socially, physically, and mentally healthy. In the other way, if she can perform her usual activity, comes for follow-up services, and if she can comprehend what clinicians have said about her health, we can say she is mentally healthy. (Female, aged 50 years)

Nearly half of participants stated that they did not know about perinatal depression, or they were not sure about it. Other respondents argued that there was no literature or documents written about perinatal depression and the available working guidelines by the government did not mention perinatal depression. Some respondents did not distinguish between perinatal and post-natal depression. One of the interviewees indicated their lack of knowledge about perinatal depression:

I do not have any idea about perinatal depression though I am a non-communicable disease officer. We are using the new non-communicable disease guideline developed by the Federal Ministry of Health and perinatal depression is not included in the guideline. (Male, aged 41 years)

There were range of understandings expressed by participants, some of whom indicated relatively limited awareness about risk factors for perinatal depression. Several respondents proposed that individual characteristics such as female sex, younger age or older age, and personal misbehaviours such as substance use, alcoholism, drug and smoking are risk factors for depression. One of the participants mentioned,

Those who are addicted to alcohol, chat, cigarettes are more depressed. After they already immersed into it, and when they could not get these substances, they would develop depression symptoms. (Male, aged 52 years).

The reason for this (depression) might not be clear but the epidemiology showed that depression is higher in females than males. This might be because women are not emotionally strong than males. (Male, aged 29 years)

Fewer than a quarter of participants proposed that stress associated with the physiological and hormonal changes happening during pregnancy and the postnatal period could lead to depression and this mostly happen in the first trimester of pregnancy. To demonstrate this, participants said:

Depression could occur during pregnancy or after birth because of some hormonal imbalances and the women would benefit from psychosocial support given by partner or any family members. (Male, aged 26 years).

...especially pregnant women, if in their early stage of pregnancy, when they feel different or start thinking about their pregnancy and the new environment after birth, they might be exposed to depression. (Male, aged 29 years)

Most participants, however, argued that the perinatal period by itself was not a risk factor for depression except for women facing additional risk factors such as early age or first-time pregnancy, cultural beliefs, economic concerns, lifestyle risks such as substance misuse, poor health status during the perinatal period, sleep problems, and/or psychosocial problems related to marital relations, limited partner and social support, or unwanted or unplanned pregnancy.

Sometimes it (depression) might happen to women when they are in low economic condition or financial struggle. Mostly, pregnant mothers who were pregnant for unwanted or unintended pregnancy are also stressed. If there are young to their age and very difficult for them to handle the pregnancy or they have other duties, they might start to feel depressed. However, I do not believe that pregnancy by itself is a risk factor for depression. (Female, aged 55 years).

… as a culture, when everybody comes to visit the mother, coffee should be served, and such gathering having repeated coffee ceremony might affect their (women) sleep frequency and quality as it is known that caffeine interferes with sleep. Taking care of their kids for long time in the night and their responsibility of leading family put these women not to have adequate sleep leading them to stress and depression. (Male, aged 29 years)

Further cultural risk factors for depression identified by participants included the lack of a person accompanying the mother during delivery.

…, for example, there is a saying after delivery called, “they leave me alone” or “she/he left me alone”. Psychosis or post psychosis might occur like this. Isn’t it? They might be tensioned for unknown reasons; can’t we say this a peripheral psychosis? (Female, aged 55 years).

While psychosocial, genetic, and biological changes related to pregnancy and childbirth were identified as risk factors, some participants stated their belief that the cause of perinatal depression is not well known.

The cause of depression could be related to delivery, pregnancy, genetics or natural, but this is one of the controversial issues for debate. (Male, aged 32 years).

Most participants described signs and symptoms of perinatal depression that fell into either physical or psychosocial categories. Physical symptoms included feeling tired, being sleepy, loss of appetite, weight gain, headache, disorganised speech, not responding, inability to speak, unable to accomplish daily activities, over-sleeping, mood changes, shivering and unconsciousness. Psychosocial symptoms proposed by participants included feelings of worthlessness, sadness or sorrow, hopelessness, stress or anxiety, loneliness or self-isolation, dissatisfaction with health services, agoraphobia, inappropriate clothing, suicidal ideation and even suicide attempts.

Mother with depression could show signs such as lethargic, not speaking correctly, unable to give their address, I know these. For example, they might not care for themselves or their foetus or infant. if they have HIV, they might not use condoms correctly. They might not be satisfied with their routine life, they might hate to do their usual activity, or they hate to speak to you. They might show feeling of worthlessness, suicide, sad, sorrow, tiredness, and they would not dress their clothes properly. (Female, aged 55 years)

One participant indicated that they were not sure whether depression symptoms were different for perinatal women than the general population and was not able to mention general signs and symptoms that everybody with depression shows.

It is not specifically to mothers, that I do not know, but I can tell you the general signs and symptoms of depression that anybody with depression could show. (Male, aged 32 years)

### Management of and interventions for perinatal depression

Participants’ knowledge or awareness about perinatal depression was not specific. Participants commonly compared the extent of depression occurring during pregnancy and after birth. Some suggested that depression occurred more commonly during pregnancy than after birth. Another argued that depression rarely occurred during pregnancy but was more common after birth.

In some of the mothers, it might occur early like during their first trimester, but most of the time, it occurs at the end of the pregnancy. After birth, it is not common, but depression might occur in a few of the mothers. (Female, aged 32 years)

Sometimes depression might occur at the time of delivery though it was not reported in our institution. However, most of the time it occurred after delivery or during the postnatal period. (Male, aged 27 years).

Furthermore, not all participants were able to identify the period by which signs and symptoms of perinatal depression might manifest:

Honestly speaking I do not really know the time by which these mothers start to show signs of depression or develop signs of depression. (Male, aged 32 years).

Participants’ attitudes towards a specific time of screening for perinatal depression is correlated with the time of occurrence of depression. Those who believed that depression is a problem of pregnancy perceived that screening should be conducted during any contact made with pregnant women during pregnancy, and they proposed the ANC visit as an appropriate time. Those who believed that depression was a problem of the postnatal period stated that depression screening should take place during the postnatal visit. In general, the best time proposed by many participants was at the time of ANC and PNC visits.

The screening should take place starting from the time of pregnancy until the postnatal period as we do not exactly know when the depression signs start to manifest. However, we give focus to postnatal depression as its prevalence is high. When we say postnatal, it includes from four to six weeks. (Male, aged 28 years).

Mainly if it (depression) should be screened, screening should be conducted during pregnancy when she comes for ANC follow up, when she comes for delivery, and thirdly after delivery when she comes for PNC follow up. (Male, aged 27 years).

Some participants indicated that screening for depression should be conducted at outpatient departments in health facilities.

I would be happy if screening could be done all the times. For example, in the outpatient department. Yet, we do not have psychiatry nurses in our health centre, and if we have one that can screen depression, this will help in reducing its burden. (Female, aged 55 years).

Other participants suggested that perinatal depression screening could be conducted during house to house visits.

The screening of depression for mothers should be started at their home by health extension workers as the urban health extension workers’ package addresses mental health issues. (Male, aged 41 years).

Disagreeing with the above, another participant working at a higher level in the healthcare system claimed that there was no evidence clearly showing that depression during pregnancy is common or that screening should be implemented.

There is nothing that says pregnant mothers are at risk of depression, and they should be assessed at this point. For example, I know that a pregnant mother who has suspected to have sexually transmitted diseases should be checked and treated after three months. But there is no study that recommended time by which depression during pregnancy should be assessed and treated. There is no screening procedure for depression in pregnancy. (Male, aged 52 years).

Two main views emerged from the analysis: (i) mothers with depression symptoms should be referred to hospitals because these are the only places where antipsychotic drugs are available, (ii) psychosocial support should be provided by health professionals, partners or families of mothers. Almost all participants recommended psychosocial support in the first place, with treatment for psychosis if the condition was severe.

For those who had depression, we provide social support by identifying possible sources of depression through psychological treatment or psychotherapy. If it (the depression) is severe enough, we provide them with psychotic drugs. And for mothers who have minor depression, their family should be informed or advised on how to provide them (the mothers) with support. (Male, aged 34 years)

### Consequences of untreated perinatal depression

Almost all participants raised concerns that untreated depression would develop into a severe mental health disorder and potentially lead to suicide.

Unless we diagnose and treat depression at an early stage, it might develop into an irreversible psychiatric problem such as dementia, which is unwanted. (Male, aged 29 years).

Most participants also suggested that untreated depression could lead to maternal suicide and death, proposing this could be through worsening to a severe form of health disorder that affects health seeking behaviour or makes women feel lonely and hopeless. Other participants suggested that depression directly leads to death because it is reported to be a major cause of death worldwide. Interviewed hospital workers stated:

Finally, if they are not treated from depression, they start to feel hopeless and, at last, go to suicide. When they had severe thought of hopelessness, they start asking about what is living for them. As they lose the meaning of life, living in this world would be nothing for them. So, they start with the idea of suicide, then they attempt and commit suicide at the end. (Male, aged 34 years).

The end consequence is death as depression by itself is a disease that cause death. (Male, aged 27 years)

The effect of perinatal depression on foetal development and birth outcomes was discussed. Participants explained the link in several ways. (a) Not using antidepressant medication correctly could affect foetal development and birth outcome; (b) genetic transmission of depression via placenta could lead the newborn baby to foetal distress and death; (c) depression could cause high blood pressure that would complicate the pregnancy and lead to abortion; and (d) depression could affect the nutritional status of the pregnant woman and foetal development.

Her foetus might have retardation, and in our culture, it is having been believed that, if the mother has depression, it also passes to the kid genetically. Based on my information or what I have heard, your foetus is healthy if you are healthy or your foetus is active if you are active. (Female, aged 55 years)

During pregnancy, a mother in severe depression might not feed herself well; if she feels unmotivated or inactive, the foetus will not develop well, its growth would be restricted, or the pregnancy might end up in abortion. (Male, aged 26 years).

Adverse effects of perinatal depression on infant health (such as malnutrition, illnesses, and death) were suggested by the participants.

The mother might die from depression or other related conditions leaving the newborn orphaned, which affects cares to be given for the newborn that leads to poor growth or death. (Male, aged 27 years).

Nearly half of the participants identified the consequences of perinatal depression on social or family disruption through reduced income, disrupted relationships, and inability to work.

She might not correctly work what she has been working because of the depression. So, she might affect her family income as depression affects her work interest and productivity. (Male, aged 52 years).

Depression affects health of the mother and this indirectly affects the family. For instance, the mother might be unable to handle her family or not well functioning in performing routine activities in the family. (Male, aged 41 years).

### Perceptions on community awareness about perinatal depression

Participants were concerned that women might seek out cultural and religious approaches to manage their depression rather than conventional health services by thinking of the disorder as evil and giving it other cultural meanings. Participants mentioned that public (community) awareness about mental health disorders, including depression, is low and people were not aware that such mental disorders are treatable.

In fact, in addition to the lack of data, in our area where we are living, culturally, mothers would not prefer to go to health facilities when such disorder is happening to them. As depression is considered evil and demonic, most of the time, perinatal women prefer to go other places for service such as spiritual places to use holy water. (Female, aged 36 years)

The other barrier is community awareness on mental health condition or depression, they do not know that this condition is treatable. (Male, aged 52 years).

### The health care system

Three sub-themes emerged from interviews under the main theme of the fragmented healthcare system: (i) perinatal mental health policy and strategy; (ii) perinatal mental healthcare system; and (iii) government capacity, readiness, and prioritisation of perinatal depression.

### Perinatal mental health policy and health care system

Most participants expressed concerns that mental health services in general were compromised. Participants tried to underline the lack of attention to mental health services in Ethiopia by highlighting the lack of mental health policies and programs that should guide government activities.

So, I can say mental health issue and concerns are not receiving much attention from the top government. For example, if you try to contact the health bureau for issues concerning mental health, nobody gives you attention and services. Even when it is related to our ward (psychiatry ward). Generally, there is a lack of attention, starting from the policy framework, curriculum, and training. (Male, aged 34 years).

The absence of clear policy frameworks and programs might also affect appropriate training and allocation of human resources for mental health. The lack of properly organised mental health structures at different levels of the Ethiopian healthcare system may also stem from the lack of a national mental health policy and related programs. Despite being the second most populous region in the country, it does not have organised teams of mental health experts or mental health specialists able to plan and establish mental health services at the regional level. One participant said:

There is no mental health focal person at the regional level. If there is no focal person, nothing would be done. But if there is a focal person, he/she can plan, deal, arrange … (Male, aged 34 years)

The general national mental health strategy developed in 2012 [50] did not specifically address mental health need of vulnerable groups such as perinatal women, peoples with disabilities, and incarcerated people. Nearly all participants confirmed that the available national mental health strategy did not specifically focus on the diagnosis and treatment of perinatal depression. The following quote demonstrates this:

We do have a general country-level mental health strategy, but it is not specified for age, sex, or specifically designed for pregnant mothers, and it is a general approach. (Male, aged 34 years)

The main barrier, agreed by almost interviewees, was the lack of an established system to prevent, screen, and treat perinatal depression. As one participant described:

The system might be challenging; for example, it would be difficult to say that clinicians in the area of ANC can know and screen depression. If you go to other health facilities or such clinics and ask how they are screening pregnant women with depression, they would tell, we use nothing. This itself can be part of the system. So, if the mother has depression during pregnancy or after delivery, she might be missed or misdiagnosed because of the lack of provision to identify the problem. And as a psychiatry clinic leader, if I want to create a system like if I want to assign a psychiatrist in ANC or PNC department to screen depression, nobody allows me, and this is part of a system too. (male, aged 28 years)

### Government capacity, readiness, and prioritisation of perinatal depression

All those interviewed agreed that there was no effective or adequate guidance for managing perinatal depression in health facilities at different levels of healthcare delivery.

Yes, I can say the Federal Ministry of Health (FMOH) has no initiative, plan, and readiness to screen, treat, prevent, and control perinatal depression in the healthcare system. So, if FMOH has no such initiatives, it isn’t very easy, or it is obvious that health structure beneath the FMOH would have no such initiative as every activity we are doing is based on the FMOH direction. (Female, aged 36 years)

Participants identified reasons for such little attention due to: (i) reduced priority of perinatal mental health; (ii) lack of knowledge about the burden and consequences of perinatal depression; (iii) lack of training of health professionals in screening depression; and (iv) high patient loads.

#### Lack of knowledge and reduced priority of perinatal mental health

Health administrators working at higher levels of the healthcare system, where policy and strategy are developed, were found to have less knowledge than those working at lower levels of the healthcare system. There also appeared to be insufficient information about the consequences of perinatal depression to make perinatal depression a priority focus of the government, as one participant working at a higher level of the health care system said:

As I told you about this, there are no separate and specific activities. The primary thing about perinatal depression is that we do not consider it as a public health problem of significance, and we do not have data about it. It has not been the forefront of public health priority threats in this region. (Female, aged 50 years)

It has been believed that perinatal depression is not a leading cause of mortality and morbidity in Ethiopia, compared with other communicable and non-communicable diseases of pregnancy and childbirth. One participant mentioned the following to show that perinatal depression is not a priority issue for the government:

Because of many other communicable and non-communicable diseases that need fast attention, perinatal depression is not given a high priority. To reduce maternal and child mortality, hypertension, obstructed labour and infections causes higher numbers of deaths than depression. As such, if we strictly work on these issues, we might bring more changes in maternal health. We are also one of the low-income countries with limited resources, and the Ministry of Health might believe that more attention should be given for such conditions than depression. As you see, due to there being many health issues in the country, the government prioritises and focuses on interventions that benefit most of the women. (Male, aged 29 years).

#### There is lack of training in screening for depression

The question about who should be responsible for screening needs to be clearly addressed in the healthcare system and enough personnel should be trained and made available in all health facilities that are expected to intervene in perinatal depression. This comes back to perinatal depression not being included as a priority health item in the country. Human resource development is a main issue for any perinatal mental health strategy and plan, but without such plans, attention given to human workforce development would be compromised. One participant working as a coordinator at a higher level of the health care system said:

Starting screening service is not easy. We do not have health professionals who trained in mental health. It needs a psychiatrist to screen and manage perinatal depression, and these professionals are minimal, including those who are in schools. So, we do not have trained professionals now, and it is challenging. (Male, aged 52 years)

#### There is high patient load

As explained in a previous section, Ethiopian health facilities always are required to treat large numbers of patients with various acute and chronic health problems. At the same time, the country is placing increased demands of health professionals in health facilities. This might affect perinatal depression screening and management because of the time required for even relatively brief consultations with perinatal women. One interviewee said:

As this is a tertiary hospital, every client comes for better service. As such, due to time limitation, it is not easy to rule out additional problems like depression. (Male, aged 29 years)

### Enablers and opportunities for perinatal depression screening services

Participants described that mental health services as being compromised and only focused on treating those who were presenting to health facilities with severe problems. They further added that there was no system for early detection and prevention of mental health disorders in the community. However, participants identified three potential opportunities or enablers that could help the Ethiopian healthcare system to start screening for depression in perinatal women and to establish its effective management. These opportunities were: the introduction of the WHO Mental Health Gap (mhGap) [51] action program; health professionals’ commitment; and simplicity of the screening program.

Participants suggested that the introduction of the mhGap initiative could potentially provide an opportunity to start and expand maternal mental health interventions in Ethiopia.

Until now, the available policy does not allow non-psychiatry health professionals to provide psychiatric services. Mental health services have been limited at the hospital level. But nowadays, because of the findings by WHO that the burden is becoming high, mental health is getting attention. One psychiatrist used to serve a population of 100,000. These days, health professionals are being trained, protocols are being under preparation, and activities have been started under MhGap initiatives to bring mental health services to health centre level. (Male, aged 52 years)

Participants hoped that screening perinatal women for depression would not be more challenging than what they currently do for everyone visiting a health facility. Health administrators mentioned that health professionals such as themselves were highly motivated to make screening available and to manage perinatal depression if the health system could be made ready for this. One participant stated that it is possible to make screening for perinatal depression available if the environment is ready:

There is no guideline and system for screening and management of perinatal depression. If there was a system, we could identify perinatal depression. I don’t think there would be a problem for us to implement if the screening program was made available. I believe there should be such a service for our women (screening, referral, treatment), I understood now. (Female, aged 37 years).

Simplicity of the screening activity was another potential enabler for instituting screening in health facilities. Participants suggested that screening would not be difficult relative to other clinical assessments that may require laboratory facilities, and additional skilled professionals. Using a brief screening tool, screening for depression might require a maximum of 15 min to implement. Similarly, additional physical space would not be required for screening because assessments could be undertaken in the same rooms where ANC and PNC services are delivered. One participant stated that health professionals are motivated and committed to undertake screening, and the only problem is lack of skill and a supportive system.

Yes, maybe we would screen and refer, this would be simple. I can see it is possible to screen pregnant and postnatal women with depression. I saw a Master’s student who did the screening in our health centre, so it is also possible as you are also doing the screening as well. (Female, aged 50 years)

## Discussion

This study explored barriers and enablers to implementing perinatal depression health services in Ethiopia. Although there was reporting of initiatives to address mental health service issues including the WHO Mental Health Gap (mhGap action program), further actions seemed to be needed to achieve effective implementation of such initiatives. For example, the current level of mental health literacy of policy makers and healthcare system leaders and the organisational context of the Ethiopian healthcare system seemed to be one of the bottlenecks for effective mhGap action program implementation [52]. Specifically, the following barriers were identified in the current study: (i) At the individual level, health administrators have little knowledge about perinatal depression risk factors, symptoms, optimal time for screening, treatment options, and the potential consequences of depression. (ii) At the socio-cultural level, there is low awareness about perinatal depression in the community, reduced health-seeking behaviours and prohibitive cultural norms; (iii) Organisational level barriers include lack of government capacity, readiness, and priority to screen and manage perinatal depression; (iv) Structural level barriers include lack of perinatal mental health policies and strategies, and transparency in the healthcare system. In addition, the study found that the introduction of the new mhGap action program, health professionals’ commitment, and simplicity of screening program could represent opportunities for or enablers of implementation of perinatal mental health services.

Health administrators’ low knowledge about perinatal depression risk factors, signs and symptoms, time of screening, health consequences and interventions are identified as individual level barriers for perinatal depression service implementation. Consistent with our findings, health administrators’ low level of knowledge in defining and conceptualising perinatal depression emerged as one of the barrier to diagnosis and treatment of perinatal depression in other studies [53, 54]. Similarly, health professionals’ low level of knowledge about signs and symptoms of perinatal depression and difficulties in identifying women with perinatal depression have been previously reported as barriers [55, 56]. This low level of knowledge about perinatal depression among health administrators and health professionals knowledge could constitute a major barrier for effective integration of maternal mental health and routine health services at the primary health care level [57]. Good mental health literacy is important for improving health, healthcare systems, and health policy [58, 59] and this should be a priority issue for health professionals working at administrative level. As such, previously conducted reviews in low- and middle-income countries have highlighted the need to build capacity of policy makers and planners to help strengthen mental healthcare systems [60, 61].

Study participants identified low community awareness about perinatal depression, health-seeking behaviours, and cultural norms as potential socio-cultural level barriers for implementation of perinatal depression healthcare services. As described by participants, many women in the community did not attend health services because cultural norms considered depression as ‘evil’ or ‘demonic’ rather than as a health problem that could be treatable. These norms are similar to cultural and community level barriers to access for mental health services identified in a systematic review and meta-synthesis of qualitative studies in the UK [34] and elsewhere [62–64]. Similarly, low community mental health literacy and a lack of models for multisectoral collaboration between traditional and religious healers were also reported as key challenges for implementing an integrated mental health care in low- and middle-income countries [65]. As such, health information on common perinatal mental health disorders should be provided for perinatal mothers in the community through health extension workers using a culturally sensitive approach. Furthermore, creation of awareness about the differences between normal pregnancy feelings and perinatal depression symptoms might be required to increase health seeking behaviour of women.

Lack of government capacity, readiness, and priority for screening and managing perinatal depression were organisational level barriers to perinatal mental health interventions discussed by study participants. High patient loads, lack of trained workforce and resources such as screening tools, guidelines, working manuals, and treatment protocols were potentially linked to lack of government capacity, readiness, and priority. These barriers should be addressed if an efficient depression screening and management program is to be established in the country. Our findings were consistent with organisational level factors reported by Smith [34] from a multi-level model of barriers to perinatal mental health interventions. Staff workloads and lack of time with health professionals for health service linkage were reported to be barriers to screening and referral for perinatal depression in high-income countries [66–68]. Furthermore, lack of training in mental health care, resources, and locally validated screening tools, together with health professionals’ negative attitudes towards screening were reported as barriers in systematic reviews of qualitative studies [69–72]. Insufficient or lack of training, unexplained long waiting times, inconsistent screening practices, and not knowing the scope of practice were mentioned as common barriers for identification and treatment of perinatal mental disorders in other systematic reviews [73, 74]. Low levels of funding have also been reported as a key challenge for sustainable mental health services because of widespread poverty and inequalities of access in low-income countries [75]. As such, it has been suggested that both perceived and established barriers associated with client or service providers should be addressed to create a suitable environment for implementing and maintaining mental health continuity of care in health facilities [76, 77].

Lack of perinatal mental health policies and strategies were proposed as structural level barriers to implementation of perinatal depression health services in Ethiopia. Lack of clarity in policies about how to screen for perinatal depression, and lack of clear pathways of care for those who had symptoms, were reported as structural level barriers to perinatal mental health interventions in a systematic review of qualitative studies [34]. The health policy of the government of Ethiopia has a central focus on preventing and treating diseases [78], but it does not address the issue of perinatal depression. Moreover, in the National Mental Health Strategy developed in 2012, only two paragraphs were devoted to reproductive mental health. There is also a lack of clarity on what should be done and how, and, generally, issues with its implementation have not been well addressed [50]. For example, a situational analysis in five low- and middle-income countries including Ethiopia revealed a lack of evidence for feasible detection and treatment strategies for mental health disorders [72]. The probable reason was a lack of priority that possibly emanated from lack of information or data on the burden and consequences of perinatal depression.

The absence of clear pathways in the healthcare system was the other most commonly discussed structural level barrier to implementation of perinatal depression services. This is consistent with structural barriers reported in a multi-level model of barriers of perinatal mental health interventions in high-income countries [34]. Perinatal women with depression symptoms in Malawi proposed that strengthening the healthcare delivery system was the most important issue to address their needs [79]. Lack of clear work structures or systems for identifying perinatal women with depression and broken referral pathways were identified as important structural barriers to perinatal mental health intervention in a systematic review [62]. In another study, structural or system level barriers to perinatal mental health implementation included complex and unclear pathways such as unlinked services, lack of continuity of care, scarcity of referral resources, and complex bureaucratic procedures [73].

Several studies have indicated that perinatal depression is both a complication [80] and/or a consequence of complications in pregnancy [81–83]. Perinatal depression could be a cause of complications that significantly affect pregnancy outcomes [76, 84, 85], child development, and higher maternal risk of subsequent psychological morbidities [72, 86–89]. Screening and providing psychotherapy to perinatal women at higher risk of depression would not significantly increase the overall cost of health expenditure in the health care system, and its benefit would outweigh the cost [90–92]. Antenatal and postnatal depression prevalences as high as 32% [93] and 34% [5], respectively have been reported in Ethiopia, and perinatal depression is documented as having multifaceted consequences for children [8, 94] and mothers [95]. In this context, the American Psychiatric Association [96], College of Obstetrics and Gynaecology [97], US Preventive Service Task Force [98], and the World Health Organization [99] have recommended screening and treatment. The effectiveness of screening and preliminary counselling interventions in preventing perinatal depression has also been documented in recent literature [90–92]. Given this evidence, it is important for the government of Ethiopia to consider the integration of perinatal depression into routine maternal health services by overcoming the current complexities [100]. The following opportunities and enablers that were discussed by study participants might help to simplify this integration.

Health administrators in this study discussed the introduction of the new mhGap action program, health professionals’ commitment, and simplicity of screening programs as enablers or facilitators for perinatal mental health service implementation. This is in line with a qualitative study that explored health professionals’ positive attitude towards the integration of mental health services as a facilitator for the acceptability of mental health services for perinatal women in Ethiopia [101]. The mhGap action program prioritises the integration of mental health services into primary care services, and maternal mental health has been considered as an essential component of this integration [51]. The mhGap initiative aims to train and use generalist nurses to diagnose and treat mental health disorders at the health centre level, which could potentially help to overcome shortage of human resources. It also aims to increase mental health awareness in the community to improve early detection and treatment. Perinatal women are more likely to be screened when health professionals are sensitive and interested, when there is continuity of care, and women are reassured that depression screening is part of a routine antenatal care [63, 102–104]. Similarly, both qualitative and quantitative studies have shown a high level of acceptance of perinatal depression screening by health providers and perinatal patients [64, 66, 105, 106]. The simplicity of screening programs adds support because any health professional can undertake screening, or the screening tool could be self-administered by mothers [107], assuming the screening tool is validated and culturally acceptable [108, 109].

There are limitations that should be born in mind during the interpretation and use of these findings for decision making. Some of the younger participants had limited experience, which could affect an in-depth understanding of health policies, programs, and strategies of the country in general and perinatal depression. Although participants were assured of the confidentiality, it is possible that potential introduction of social desirability bias existed with some participants being concerned of their responses being communicated to higher officials. There was no relationship between the study participants and the researcher prior to the study but there could be researcher bias associated with prior assumption about the research topic. However, to reduce these issues, the interviewer assured the confidentiality of the information they provided, interview was undertaken in private office, and clarified that the role of the interviewer was interviewing and would not have any connection with the health delivery system. At most, the researcher restricted himself from using leading probes toward his assumptions about the problem during data collection and he only analysed and presented information generated from the data. Despite these limitations, this is the first attempt to explore barriers to and enablers of perinatal depression service implementation in Ethiopia. As such, the findings will therefore be useful to health planners, researchers, partners and, ultimately, perinatal women and their infants.

## Conclusions and recommendations

This study aimed to identify barriers and enablers to the provision of perinatal mental health services in Ethiopia. The study identified health administrators’ low literacy about perinatal depression as an individual level barrier; community low awareness, health-seeking behaviours and cultural norms about perinatal depression as socio-cultural level barriers; lack of government capacity, readiness, and priority for screening and managing perinatal depression as organisational level barriers; and lack of perinatal mental health policies, strategies, and healthcare systems as structural level barriers for perinatal mental health service implementations in Ethiopia. On the other hand, introduction of the new mhGap action program, health professionals’ commitment to providing effective services, and simplicity of screening programs were identified as potential enablers or facilitators for perinatal mental health service implementation. Building the capacity of policy makers and planners should be a first step in mental healthcare system building related to perinatal depression in Ethiopia. Strengthening the mental healthcare system and governance to produce clear mental health policies, programs, strategies, structures, and legislative framework is mandatory for effective integration of maternal mental health care with routine maternal health services in Ethiopia.

## .

## Data Availability

The datasets used and/or analysed during the current study available from the corresponding author on reasonable request as this is part of a PhD work.

## Abbreviations

WHO: World Health Organization
DSM-IV: Diagnostic and Statistical Manual of Mental Disorder
AND: Antenatal Depression
EPDS: Edinburgh Postnatal Depression Scale
SBRC: Social and Behavioral Research Ethics Committee
